# Pregnancy-induced Cushing syndrome: diagnosis and management of a rare endocrine disorder

**DOI:** 10.1210/jcemcr/luag134

**Published:** 2026-06-02

**Authors:** Ronit Koren, Efrat Markus, Yifat Wiener, Yona Greenman, Shlomit Koren

**Affiliations:** Department of Internal Medicine A, Shamir Medical Center (Assaf Harofeh), Be'er Ya'akov, Zerifin 70300, Israel; Gray Faculty of Medicine, Tel Aviv University, Tel Aviv 6997801, Israel; Endocrine Institute, Shamir Medical Center, Be'er Ya'akov, Zerifin 70300, Israel; Gray Faculty of Medicine, Tel Aviv University, Tel Aviv 6997801, Israel; Division of Maternal Fetal Medicine, Department of Obstetrics and Gynecology, Be'er Ya'akov, Zerifin 70300, Israel; Gray Faculty of Medicine, Tel Aviv University, Tel Aviv 6997801, Israel; Institute of Endocrinology, Metabolism, Diabetes and Hypertension, Tel Aviv-Sourasky Medical Center, Tel Aviv 64239, Israel; Gray Faculty of Medicine, Tel Aviv University, Tel Aviv 6997801, Israel; Endocrine Institute, Shamir Medical Center, Be'er Ya'akov, Zerifin 70300, Israel

**Keywords:** pregnancy-induced Cushing syndrome, metyrapone, aberrant adrenal receptors

## Abstract

Pregnancy-induced Cushing syndrome (PICS) is an exceptionally rare form of adrenocorticotropin (ACTH)-independent Cushing syndrome (CS) caused by aberrant expression of luteinizing hormone (LH) and/or human chorionic gonadotropin (hCG) receptors in adrenal cortical cells. To date, only a small number of cases have been reported, most with adrenal abnormalities on imaging. We describe a 25-year-old pregnant woman who presented at 26 weeks’ gestation with new-onset hypertension and overt features of hypercortisolism, including severe acne, hirsutism, dorsocervical fat pad, and extensive violaceous striae. Biochemical evaluation confirmed severe ACTH-independent CS with markedly elevated 24-hour urinary free cortisol, elevated serum cortisol with loss of diurnal variation, elevated midnight salivary cortisol, and suppressed ACTH levels. Pituitary, adrenal, and ovarian imaging were unremarkable. The patient was successfully managed with metyrapone until delivery, with favorable maternal and neonatal outcomes. Postpartum resolution of hypercortisolism and a positive hCG stimulation test confirmed the diagnosis of PICS. This case highlights the diagnostic complexity of PICS and underscores the role of timely medical therapy and multidisciplinary management.

## Introduction

Endogenous Cushing syndrome (CS) is a rare disorder associated with substantial morbidity and mortality. Its occurrence during pregnancy is exceedingly uncommon, largely due to the suppressive effects of hypercortisolism on the hypothalamic-pituitary-gonadal axis. Between 1952 and 2015, approximately 220 cases of CS in pregnant women were reported [1]. Most cases (∼60%) were of adrenal origin, followed by pituitary disease in about one-third of cases. Rare etiologies include adrenal carcinoma, nodular adrenal hyperplasia, and ectopic adrenocorticotropin (ACTH) secretion [2].

Pregnancy-induced Cushing syndrome (PICS) represents an exceptionally rare entity of ACTH-independent CS. It results from aberrant adrenal expression of luteinizing hormone (LH) and/or human chorionic gonadotropin (hCG) receptors on adrenal cortical cells, leading to hormone-driven cortisol synthesis, cellular proliferation, and adrenal hyperplasia [3]. To date, only 16 cases of PICS have been described, the majority of which demonstrated adrenal adenomas or hyperplasia on imaging [4].

We report a unique case of PICS without radiographic adrenal abnormalities, successfully managed medically throughout pregnancy.

## Case presentation

A 25-year-old woman presented at 26 weeks of gestation with newly diagnosed hypertension. Her medical history was notable for epilepsy, well controlled with lamotrigine. She denied any exogenous steroid use, and her family history was unremarkable.

On examination, her blood pressure was 150/100 mm Hg and pulse rate was 100 beats per minute. She was afebrile. Physical examination revealed severe acne, hirsutism, a dorsocervical fat pad (“buffalo hump”), and extensive violaceous striae involving the abdomen, arms, and legs. The patient reported a weight gain of 30 kg during pregnancy. The glucose challenge test was abnormal at 167 mg/dL (9.3 mmol/L).

## Diagnostic assessment

**Figure 1:**
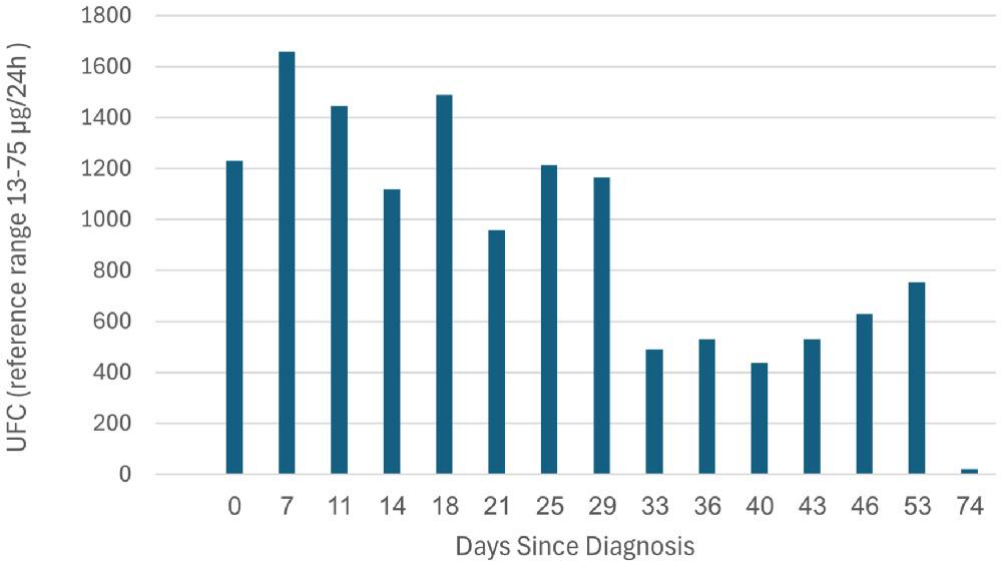
Shown are 24-hour urinary free cortisol levels according to week post diagnosis. *During the first 5 weeks metyrapone treatment was initiated and titrated from 500 to 2000 mg/day, and was stopped 9 weeks post diagnosis (after delivery).

Laboratory evaluation demonstrated markedly elevated urinary free cortisol (UFC) of 1659 µg/24 hours (SI: 4578 nmol/24 h) (reference range, 13-75 µg/24 h [SI: 35-207 nmol/24 h]) (Fig. 1). Serum cortisol was elevated at 47.5 µg/dL (SI: 1313 nmol/L) (reference range, 6-23 µg/dL [SI: 138-635 nmol/L]) early in the morning and remained unsuppressed at midnight (47.1 µg/dL [SI: 1300]) (reference range <5 µg/dL [SI: <138 nmol/L]). Late-night salivary cortisol (LNSC) levels were markedly elevated (>3 µg/dL (SI: >82 nmol/L) (reference range <0.12 µg/dL [SI: <3.3 nmol/L]), acknowledging the absence of pregnancy-specific reference ranges. Plasma ACTH was suppressed at 1.5 pg/mL (SI: 0.33 pmol/L) (reference range, 7.2-63 pg/mL [SI: 1.58-13.87 pmol/L]). Dehydroepiandrosterone sulfate was low at 73.7 µg/dL (SI: 2.03 µmol/L) (reference range, 98.8-340 µg/dL [SI: 2.68-9.23 µmol/L]). The patient presented with hypokalemia, potassium levels of 2.9 mEq/L (SI:2.9 mmol/L) (reference range, 3.5-5 mEq/L [SI: 3.5-5 mmol/L]), direct renin level was elevated at 432 mIU/L (2.8-39 mIU/L), and aldosterone level was suppressed at 1.33 ng/dL (SI: 37 pmol/L) (reference range, 1.77-23.2 ng/dL) [SI: 49-644 pmol/L]). The suppressed aldosterone level may be explained by mineralocorticoid receptor activation due to severely elevated cortisol levels, and the hypokalemia resulting in negative feedback on the renin-angiotensin-aldosterone system. After the correction of potassium levels, aldosterone increased to 6 ng/dL (SI: 167 pmol/L).

**Figure 2:**
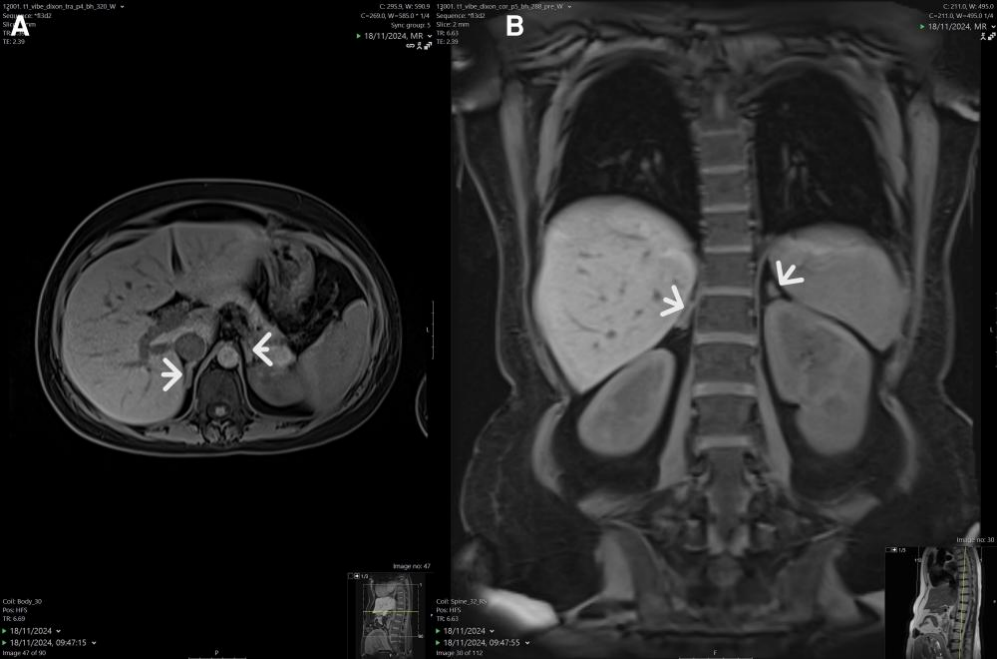
T1-weighted abdominal magnetic resonance imaging at the level of the adrenal glands (arrows): A, axial plane; B, coronal plane.

Pituitary magnetic resonance imaging (MRI) demonstrated no adenoma. Abdominal MRI showed normal-appearing adrenal glands (Fig. 2), and pelvic ultrasound revealed no ovarian masses.

## Treatment

Based on the presumptive diagnosis of PICS, treatment with metyrapone was initiated at a twice-daily dosing schedule and gradually titrated to a total dose of 2 g/day, administered in 4 divided doses at the higher dose.

Adjunctive treatments included unfractionated heparin for deep vein thrombosis prophylaxis, vitamin D and calcium supplementation, insulin detemir for gestational diabetes, labetalol and amlodipine for hypertension, and *Pneumocystis jirovecii* pneumonia prophylaxis with trimethoprim-sulfamethoxazole due to severe hypercortisolism.

Clinical improvement followed, with stabilization of blood pressure and glycemic control. Amlodipine was subsequently discontinued. Prior to delivery, biochemical improvement was noted, with reductions in morning serum cortisol to 32 µg/dL (SI: 883 nmol/L), UFC to 530 µg/24 h (SI: 1460 nmol/24 h), and LNSC to 0.59 µg/dL (SI: 16.3 nmol/L).

## Outcome and follow-up

A multidisciplinary team involving endocrinologists, obstetricians, maternal-fetal medicine specialists, pediatricians, and pediatric intensive care specialists elected to perform cesarean delivery at 34 + 5 weeks of gestation. Intraoperatively, parenteral labetalol was required for blood pressure control. A 2-kg neonate, appropriate for gestational age, was delivered.

Hydrocortisone replacement therapy was initiated for presumed neonatal adrenal insufficiency, later confirmed by ACTH stimulation testing (baseline cortisol was 0.33 µg/dL [SI: 9 nmol/L], 2.38 µg/dL [SI: 65 nmol/L] after 30 minutes, and 2.34 µg/dL [SI: 64 nmol/L] after 1 hour). Full recovery of the hypothalamic-pituitary-adrenal axis was documented 5 months after delivery (cortisol levels of 25.6 µg/dL [SI: 698 nmol/L] 1-hour post-ACTH stimulation). At 7 months the infant demonstrated normal physical and neurologic development, with age-appropriate height and weight.

Post partum, metyrapone and all adjunctive therapy were discontinued within 3 weeks. Follow-up imaging, including adrenal computed tomography scan and pituitary MRI, remained normal. Bone mineral density testing revealed mild lumbar osteopenia. One-month post partum, maternal morning serum cortisol levels were 19.1 µg/dL (SI: 527 nmol/L), UFC levels were 19.9 µg/24 h (SI: 55 nmol/24 h), and LNSC were below 0.11 µg/dL (SI: <3 nmol/L).

An hCG stimulation test performed 3 months post partum (13 000 IU of hCG was administered subcutaneously, and serial blood samples were collected for cortisol and ACTH measurements) demonstrated a marked increase in cortisol and UFC, with concomitant ACTH suppression, confirming hCG-dependent cortisol secretion consistent with PICS (Table 1).

**Table 1:** Human chorionic gonadotropin stimulation test

Parameters	Value, conventional units	Value, SI units	Reference range, conventional units (SI units)
UFC, baseline	28 µg/24 h	77 nmol/24 h	13-75 µg/24 h (SI: 35-207 nmol/24 h)
UFC, 48 h	293 µg/24 h	808 nmol/24 h	13-75 µg/24 h (SI: 35-207 nmol/24 h)
Morning cortisol, baseline	15.6 µg/dL	431 nmol/L	6-23 µg/dL (SI: 138-635 nmol/L)
Morning cortisol, 24 h	32.4 µg/dL	893 nmol/L	6-23 µg/dL (SI: 138-635 nmol/L)
Morning cortisol, 48 h	34 µg/dL	939 nmol/L	6-23 µg/dL (SI: 138-635 nmol/L)
Morning cortisol, 72 h	31.3 µg/dL	863 nmol/L	6-23 µg/dL (SI: 138-635 nmol/L)
ACTH, baseline	14.4 pg/mL	3.17 pmol/L	7.2-63 pg/mL (SI: 1.58-13.87 pmol/L)
ACTH, 24 h	<5 pg/mL	<1.1 pmol/L	7.2-63 pg/mL (SI: 1.58-13.87 pmol/L)
ACTH, 48 h	<5 pg/mL	<1.1 pmol/L	7.2-63 pg/mL (SI: 1.58-13.87 pmol/L)
ACTH, 72 h	<5 pg/mL	<1.1 pmol/L	7.2-63 pg/mL (SI: 1.58-13.87 pmol/L)
hCG, baseline	<3 IU/L	<3 IU/L	Nonpregnant <3 IU/L (SI: <3 IU/L)
hCG, 24 h	166 IU/L	166 IU/L	Nonpregnant <3 IU/L (SI: <3 IU/L)
hCG, 48 h	127 IU/L	127 IU/L	Nonpregnant <3 IU/L (SI: <3 IU/L)
hCG, 72 h	82 IU/L	82 IU/L	Nonpregnant <3 IU/L (SI: <3 IU/L)
LH, baseline	7.5 IU/L	7.5 IU/L	Follicular phase 1.9-12.5 IU/L (SI: 1.9-12.5 IU/L)
LH, 24 h	5.4 IU/L	5.4 IU/L	Follicular phase 1.9-12.5 IU/L (SI: 1.9-12.5 IU/L)
LH, 48 h	5.8 IU/L	5.8 IU/L	Follicular phase 1.9-12.5 IU/L (SI: 1.9-12.5 IU/L)
LH, 72 h	3.7 IU/L	3.7 IU/L	Follicular phase 1.9-12.5 IU/L (SI: 1.9-12.5 IU/L)

Abbreviations: ACTH, adrenocorticotropin; hCG, human chorionic gonadotropin; LH, luteinizing hormone; UFC, urinary free cortisol.

The patient declined genetic testing; hence the diagnosis of primary pigmented nodular adrenocortical disease (PPNAD) could not be excluded. She was counseled on the options of adrenalectomy or surrogacy for future pregnancies but elected to pursue conservative medical management for subsequent pregnancies. Preconception counseling was strongly advised for the early identification of clinical and biochemical features suggestive of CS. Prompt initiation of metyrapone therapy in future pregnancies may mitigate the substantial morbidity associated with this condition.

## Discussion

PICS is an exceptionally rare disorder with only 16 published cases. Although most reported cases demonstrated adrenal adenomas or hyperplasia, adrenal imaging may be normal, as in the present case. Four cases reported an hCG stimulation test. In 3 of them postpartum cortisol levels significantly increased after hCG administration. Seven cases reported metyrapone use during pregnancy [4-19].

Management of CS during pregnancy is challenging due to limited evidence, absence of approved pharmacologic therapies, and physiological changes in cortisol regulation during pregnancy. Estrogen-induced increases in corticosteroid-binding globulin elevate total cortisol levels, while placental ACTH and corticotropin-releasing hormone increase free cortisol levels in serum, saliva, and urine, reaching values that may overlap with those detected in CS. The absence of well-defined pregnancy-specific reference ranges complicates biochemical interpretation and treatment titration.

Active CS during pregnancy is associated with increased maternal risks, including diabetes, hypertension and preeclampsia, thromboembolism, and operative delivery, as well as fetal complications such as prematurity, growth restriction, neonatal hypoglycemia, and transient adrenal insufficiency [20].

Aberrant adrenal expression of G protein–coupled receptors has been identified in ACTH-independent CS, including LH/hCG receptors, glucose-dependent insulinotropic polypeptide receptors, β-adrenergic receptors, vasopressin receptors, glucagon receptors, and serotonin receptors [5, 8]. Receptor binding with activation of cyclic adenosine monophosphate signaling leading to cortisol overproduction and adrenal hyperplasia represents a well-described mechanism of ACTH-independent hypercortisolism [5]. Although genetic or histologic confirmation was not available in the present case, postpartum resolution of hypercortisolism and a positive hCG stimulation test provided strong functional evidence supporting the diagnosis.

Although no pharmacologic therapies are formally approved for CS during pregnancy, metyrapone is the most commonly used agent and is considered first-line therapy when treatment is required [2, 14, 18, 21, 22].

According to the latest Pituitary Society guidelines, the use of cabergoline or metyrapone should be considered during pregnancy [23]. Since cortisol levels are higher than normal, different cutoff values for the titration of treatment are recommended, such as 1.5 times the upper limit of normal. In this case report, metyrapone was successfully used with no adverse events.

Fetal morbidity and mortality are high in women with CS. According to a systematic review of cases from 1952 to 2015, the main predictor of fetal loss was the etiology of hypercortisolism with an odds ratio of 4.7 for PICS vs Cushing disease. Fetal morbidity was related mainly to timing of diagnosis (during vs prior to pregnancy) [1].

Given the limited number of reported cases, this case adds important insights to the existing literature on PICS. Normal-appearing adrenal glands should not exclude the diagnosis, and a postpartum hCG stimulation test, which is relatively simple to perform, can provide supportive functional confirmation. In addition, this case contributes to the growing body of evidence supporting the use of metyrapone during pregnancy.

In summary, this case illustrates the complexity of managing PICS and highlights the importance of a multidisciplinary approach for shared decision-making regarding treatment, maternal and neonatal monitoring, delivery, postnatal care, and final diagnosis.

## Learning points

- Pregnancy-induced CS is an exceptionally rare and diagnostically challenging cause of ACTH-independent hypercortisolism.
- Normal adrenal imaging does not exclude PICS.
- Medical therapy with metyrapone, while used with caution, may be effective and well tolerated during pregnancy when carefully monitored, especially for maternal hypertension and neonatal hypocortisolism.
- Multidisciplinary management is essential to optimize maternal and neonatal outcomes.

## Data Availability

Data sharing is not applicable to this article as no datasets were generated or analyzed during the current study.

## Abbreviations

ACTH: adrenocorticotropin
CS: Cushing syndrome
hCG: human chorionic gonadotropin
LH: luteinizing hormone
LNSC: late-night salivary cortisol
MRI: magnetic resonance imaging
PICS: pregnancy-induced Cushing syndrome
UFC: urinary free cortisol
